# Time to recovery and determinants of severe acute malnutrition among 6–59 months children treated at outpatient therapeutic programme in North Gondar zone, Northwest Ethiopia: a prospective follow up study

**DOI:** 10.1186/s13052-019-0732-9

**Published:** 2019-11-04

**Authors:** Worku Nigussu Mamo, Terefe Derso, Kassahun Alemu Gelaye, Temesgen Yihunie Akalu

**Affiliations:** 1 Abrhajira Primary Hospital, West Gondar Zone, Ethiopia; 2 Department of Human Nutrition, Institute of Public Health, College of Medicine and Health Sciences, University of Gondar, Gondar, Ethiopia; 3 Department of Epidemiology and Biostatistics, Institute of Public Health, College of Medicine and Health Sciences, University of Gondar, Gondar, Ethiopia

**Keywords:** SAM, Time to recovery, North Gondar

## Abstract

**Background:**

Despite the available interventions to tackle nutritional problems, there is scarce information on time to recovery and its determinants among children with SAM in Ethiopia.

**Objective:**

This study was aimed at finding the time to recovery and determinants among 6–59 months children with severe acute malnutrition treated at an outpatient therapeutic programme in North Gondar zone, northwest Ethiopia.

**Methods:**

Facility based prospective follow up study was conducted from March 24 to May 24, 2017. A total of 408 children with the age of 6–59 months were included in the study**.** Structured interviewer administered questionnaire was used. Anthropometric measurements were conducted every week. The median time of recovery, Kaplan Meier (KM) curve, and log rank test were computed. Both bi-variable and multivariable Cox regression model was fitted. To establish an association between time to recovery and its determinants 95% confidence interval (CI) and *p*-value < 0.05 were used. Proportional hazard assumption was checked graphically and using Schoenfeld residual test.

**Results:**

Out of 389 children, 254 (65.3%) recovered. The median time to recovery was 38.5 ± IQR of 14 days**.** Children with diarrhoea AHR = 0.81 with 95% CI (0.73, 0.99), children taken amoxicillin AHR = 2.304 with 95% CI (1.68–3.161), and had vomiting at admission AHR = 0.430 with 95% CI (0.205, 0.904) were significant predictors of time to recovery.

**Conclusions and recommendations:**

The overall time to recovery has not met the minimum sphere international standard which was lower than 75%. It is advisable to give emphasis to patients with diarrhoea and vomiting.

## Introduction

Severe acute malnutrition (SAM) is defined as, a weight-for-height measurement of ≤70%, weight for height Z score < 3 Standard deviation (SD), presence of bilateral pitting edema, and/or a mid-upper-arm circumference of < 110 mm (MUAC) in children age 6–59 months [1].

According to global report acute malnutrition is a major cause of death among under-five children. Globally, in 2011 an estimated of 2 million children suffered from SAM. Out of them more than 80% were treated in four sub-Saharan Africa countries (Ethiopia (27.9%), Niger (27.6%), Somalia (15.4%), and Democratic Republic of Congo (14.5%) [2]. According to current estimates, approximately 17 million children under the age of 5 years were suffer from SAM and nearly two-thirds were from Southeast Asia and sub-Saharan Africa [3]. According to 2016 EDHS report, children who were wasted <− 2 SD were 9.9%. Similarly, the prevalence of severe wasting in Ethiopia and Amhara region was 2.9 and 2.2%, respectively [4].

SAM has a high case fatality rate despite use of available management protocols [5]. According to a systematic review and meta-analysis from low and middle income countries on the treatment of severe and moderate acute malnutrition the case fatality rate for inpatient treatment of SAM using World Health Organization (WHO) protocol ranged from 3.4 to 35% [5].

According to previous studies there are several factors contributing for time to recovery among SAM patients. Low weight gain, diarrhoea [6, 7], education and training of health worker [8], antibiotics [9], rickets [10, 11], pneumonia [11], malnutrition status [1], stabilization centers [1], age [12], put on anti-biotic (Amoxicillin) [13], sharing of ready to use therapeutic food (RUTF) [14], being vaccinated [15], children receiving water treatment [16] and poor appetite [17] were important predictors of time to recovery among SAM patients.

Despite the use of available guidelines for management of SAM, co-morbidities and poor treatment outcomes are still observed in therapeutic feeding centers [11]. However, studies on time to recovery were very limited in Ethiopia including the study area. Therefore, this study aimed at determining the time to recovery and its predictors among children with SAM in North Gondar zone, Ethiopia.

## Methods and materials

### Study design and setting

Health facility based prospective follow up study was conducted from March 24 to May 24, 2017. The study was conducted in five districts of North Gondar zone including: Dabat, Wogera, Gondar zuriya, West Armachiwo, and Tach Armachiwo districts. Seventeen health centers and 31 health posts provided OTP service in the selected health facilities.

### Population, sample size, and sampling procedure

All SAM children between 6 and 59 months and admitted in the selected health facilities were included in the study. Sample size was determined using study conducted from Enderta district, Tigray, Northern Ethiopia, 2012 [18]. So, we calculate the sample size by medcalc©version 18.11.3 survival analysis (logrank test) at http://www.medcal.org [17]. Adding 5% non response rate and design effect of 1.5 the final sample size was 408 (Table 1).

**Table 1:** Sample size determination using logrank test by medcalc©version 18.11.3

Variables		Cured	Censored	% of outcome	AHR	Log rank	*p*-value	Total event needed
Distance of HI from residence	< 2 h	196	46	72.9	1.48	19.3	< 0.001	258
	> 2 h	55	31	65.5	1			
Storage of drinking water	Bucket	193	35	84.6	1.51	21.8	0.008	101
	Pot	62	42	59.6	1			
Sex of the child	Male	124	34	78.4	1.30	12.6	0.043	25
	Female	13	43	23.2	1			
Age of the child on OTP	< 18 months	176	39	81.8	1.20	18.3	0.259	72
	> 18 months	79	81	49.5	1			
Wt/Ht at admission	< 60%	37	171	17.8	1	27.1	0.001	24
	60%	218	60	78.4	1.87			
Way food preparing for < 5 children	Separately for them	178	28	86.4	1.24	5.9	0.117	95
	Together with adult	77	49	61.1	1			

A multi-stage random sampling technique was applied. Among 22 districts 5 were selected by using simple random sampling technique. In these 5 districts, 48 health facilities had been provided OTP services. Only health centers and health posts running OTP service in the selected districts were included in the study.

### Data collection tool and procedure

A structured questionnaire was developed in English and it translated to the local Amharic language and back translated to English language to check its consistency. Structured interviewer administered questionnaire was used to collect information from each study participant. Mothers or caregivers of the selected children were interviewed. Anthropometric measurements and physical examination were used to collect data from study participants. Body weight was measured using a 25 kg hanging spring scale to the nearest 0.1 kg for children below the age of 3 years. For children less than 85 cm, the measuring board was placed on the ground and read to the nearest 0.1 cm in the recumbent position. On the other hand, for children more than 85 cm, the measuring board was fixed where the ground is level by standing position and read to the nearest 0.1 cm. Similarly, MUAC was measured on the left upper arm of a child and its value was recorded to the nearest 1 mm. Measurements like medical complications and presence of bilateral pitting edema were recorded on admission and at follow-up on a standard individual treatment card. Appetite test was conducted every week in a quite environment on each visit for child enrolled in the program. A child was said to pass the appetite test when she or he was able to consume the amount of RUTF recommended for her or his body weight. Children who failed the appetite test in any visit were referred to inpatient care. At admission, the data collectors assessed the degree of pitting edema, hydration, dysentery, diarrhoea, anemia, and other signs of infections. Each participant on OTP was visiting to their closest site weekly to receive food and a medical assessment. During every visit, the child was examined and given a weekly supply RUTF. At each follow-up visit weight of the child, existence/ extent of pitting edema, presence of disease, drugs prescribed and outcome (death, discharge cured, default, or transfer) had been recorded on patient treatment cards and in the programme register.

Two days of training on the objectives of the study and how to interview, measure, and fill the questionnaire was given to the data collectors and supervisors before the actual data collection. Forty eight BSC nurses who took training on OTP and currently working in OTP sites were recruited to collect the required information. The principal investigator and three health extension workers supervised the data collection process. Besides, the data collection tool was pretested with 20 (5%) of samples in Dembia district. Lastly, cleaning was done on daily basis and timely feedback was communicated to the data collectors.

### Measurements and study variables

Time to recovery, the outcome variable of this study was defined as a time from admission date to discharge date while the child is cured. Time to recovery was measured by subtracting the date of admission from the discharge date. Therefore, children who were reached the discharge criteria means (W/L > =85% or W/H > =85% on more than one occasion for children with Marasmus, and if edema was disappeared regardless of their body weight status within 14 days for kwashiorkor cases. Whereas, children who were admitted and treated at OTP and discharged out other than cured like transfer out, unknown, defaulter, death, or non-response and still they were in a program were censored. Socio-demographic variables: age of the mother/caregiver, sex of the child, marital status, residence, occupational status of mother’s or care giver, relation to child, ethnicity, income, educational status of mother’s or care giver; Maternal and child health related factors: child breastfeed status, deworming, bed net utilization, type of admission, routine medication during admission and follow up, medical problems during follow-up; Hygiene and sanitation factors: Source of drinking water, Latrine, housing condition were assessed. Average length of stay was defined as sum of number of days for each recovered patients per total number of patients in a program. Weight gain (g/kg/day): was defined as an average weight (in gram) increase for every Kg of body weight of the child per day.

### Data processing and analysis

Data were entered into Epi-Data version 3.1 and exported to STATA 14 for analysis. Descriptive measures like percentages and median were used to describe categorical variables and continuous variables, respectively. Other descriptive measure like life-table was computed. Proportional Hazard Assumption (PHA) was checked both graphically and using Schoenfeld residual tests. Cox regression model was computed for both bi-variable and multivariate analysis and final results were taken as significance at 5% level of significance. Adjusted hazard ratio (AHR) with its respective 95% confidence interval (CI) was reported to show the strength of association.

#### Socio-demographic characteristics of mothers/caregivers

A total of 408 patients were included in the study with a response rate of 95.34%. Mean age of the mother/caregiver was 31.25 with SD of ±9.8. Among admitted patients at OTP, 65.04% were from urban residence. Majority, 238 (61.18%) were housewives. About three-fourth of respondents were illiterate **(**Table 2**)**.

**Table 2:** Socio-demographic and economic characteristics of the mothers/guardians of children who were admitted at OTP in North Gondar, northwest Ethiopia, 2017 (*n* = 389)

Variables	Frequency	Percentage(%)
Residence		
Urban	253	65.04
Rural	136	34.96
Marital status		
Married	301	77.38
Single	56	14.39
Divorced	19	4.88
Widowed	13	3.35
Mother’s/care givers occupation		
House wife	238	61.18
Government employee	98	25.19
Farmer	18	4.63
Merchant	26	6.68
Others	9	2.32
*mother’s/care giver education*		
Illiterate	288	74.04
Literate	101	25.96
Relation to child		
Mother	283	72.75
Sibling	42	10.79
Caregiver	14	3.62
Grand mother	42	10.79
Other	8	2.05

#### Maternal and child related factors

About 311 (80.21%) were still breastfeeding their child at the time of data collection. About one-third (*33.16%*) of the children were fully immunized and 140 (36.00%) were not yet vaccinated. Regarding with merits of breastfeeding nearly one-fourth (25.96%), 164 (42.16%), 100 (25.71%) respond as breastfeeding is important for child growth, child health, and child food, respectively. About 70% children initiated complementary feeding at 6 months **(**Table 3**)**.

**Table 3:** Maternal health practice during treatment of severe acute malnutrition among children at OTP in North Gondar, northwest Ethiopia, 2017 (*n*=389)

Variables	*Frequency*	*Percentage (%)*
Child breastfeeding status		
No	77	19.79
Yes	311	80.21
Immunization		
Fully immunized	129	33.16
Partially immunized	120	30.84
Not yet vaccinated	140	36.00
Merits of breastfeeding for mother and child		
For child growth	101	25.96
For child health	164	42.16
For child food	100	25.71
For comfort	19	4.88
Other**	5	1.29
Starting period of complementary foods		
0–3 months	17	4.37
4–5 months	44	11.32
At 6 months	268	68.89
> = 7 months	30	7.71
I don’t know	30	7.71

I don’t know* not checked about his/her breastfeeding status during interviewed.

Other** the merits for mental health, for skill, knowledge

#### Health service utilization

Almost a quarter of children had medical related problems 2 weeks prior to the survey. Out of the total admissions, a total of 355 (91.26 %) were newly admitted patients, 6.17 % transferred in and 10 (2.57%) were readmitted. Among the total admitted cases, 9.3 had edema, no danger sign was looked, and a few had symptoms of diarrhea (11.82%), vomiting (3.09%), and cough (2.57%) at admission. At admission, 67.9%, 24.7%, and 13.6 % were took amoxicillin, measles, and vitamin A capsule, respectively **(**Table 4**).**

**Table 4:** Health service utilization of children who were admitted at OTP in North Gondar, northwest Ethiopia, 2017 (*n*=389)

Variables	Frequency	Percentage(%)
Types of admission		
New	355	91.26
Transferred in	24	6.17
Readmitted	10	2.57
Routine medication		
Amoxicillin		67.86
Folic acid		1.03
Measles		24.68
Albendazol/mebendazol	264	23.39
Vitamin A supplementary	264	13.62
Anti-Malarial drugs	4	2.06
	96	
	91	
	53	
	8	
Diarrhea at admission		
Yes	46	11.82
No	343	88.18
Vomiting at admission		
Yes	12	3.09
No	377	96.91
Cough at admission		
Yes	10	2.57
No	379	97.43
Diarrhea during follow up		
Yes	8	2.06
No	381	97.94
Fever during follow up		
Yes	9	2.31
No	380	97.69
Vomiting during follow up		
Yes	9	2.3
No	380	97.7
Cough during follow up		
Yes	25	6.43
No	364	93.57

### Clinical characteristics of children

Majority, 90.75% patients had sign of Marasmus. During follow-up children with SAM complicated with respiratory distress 6.68%, acute diarrhea 17.48%, and hyperthermia 2.84% and anemia accounts 1.03%. Children with severe acute malnutrition checked for any problems and only 3.86% of children reassured during follow-up **(**Table 5**)**.

**Table 5:** Clinical characteristics of children who were admitted at OTP in North Gondar, northwest Ethiopia, 2017(*n* = 389)

Variables	Frequency	Percentages
Sign of malnutrition		
Marasmus	353	90.75
Kwashiorkor	36	9.25
Complications during follow-up		
Respiratory distress	26	6.68
Acute diarrhea	68	17.48
Hyperthermia	11	2.84
Anemia	4	1.03
Admission MUAC		
< 110 mm	235	63.34
≥110 mm	154	36.66
Appetite test done at any visit consistently		
Yes	379	97.43
No	10	2.57
For any problems during follow up		
Action taken	15	3.86
Not Action taken	299	76.86
Not assessed at all	75	19.28

### Treatment outcomes of children with severe acute malnutrition

From the total study subjects, 254 (65.3%) successfully recovered from SAM within the first 8 weeks of treatment and 135 (34.7%) were censored. Among SAM children 4 (1.03%) didn’t know their status. A total of 39 (10.02%) patients were transferred out to nearby health facility, and 2 (0.51%) cases were referred to inpatient due to complications during the follow-up visits. About 17 (4.37%) cases were defaulters. The average rate of weight gain among recovered children was 5.814 g/kg/day (±2.2833).

### Survival analysis

The participants were followed for a total of 13,104 person days (35.9 person years) observation. The median time to recovery was (38.5 ± IQR 14 days). The probability of survival at 4th, 5th, 6th, 7th, 8th,and 9th weeks were 85.6, 55.2, 35.3,13.1, 5 and 2.1%, respectively.

The proportional hazard assumption was checked by the Schoenfeld residual global test, and *p*-value (*p* = 0.1464). So, proportional hazard assumption was met. Goodness of fit for the fitted model was also performed using the Cox Snell residual test and showed that the model was adequate.

**Fig. 1:**
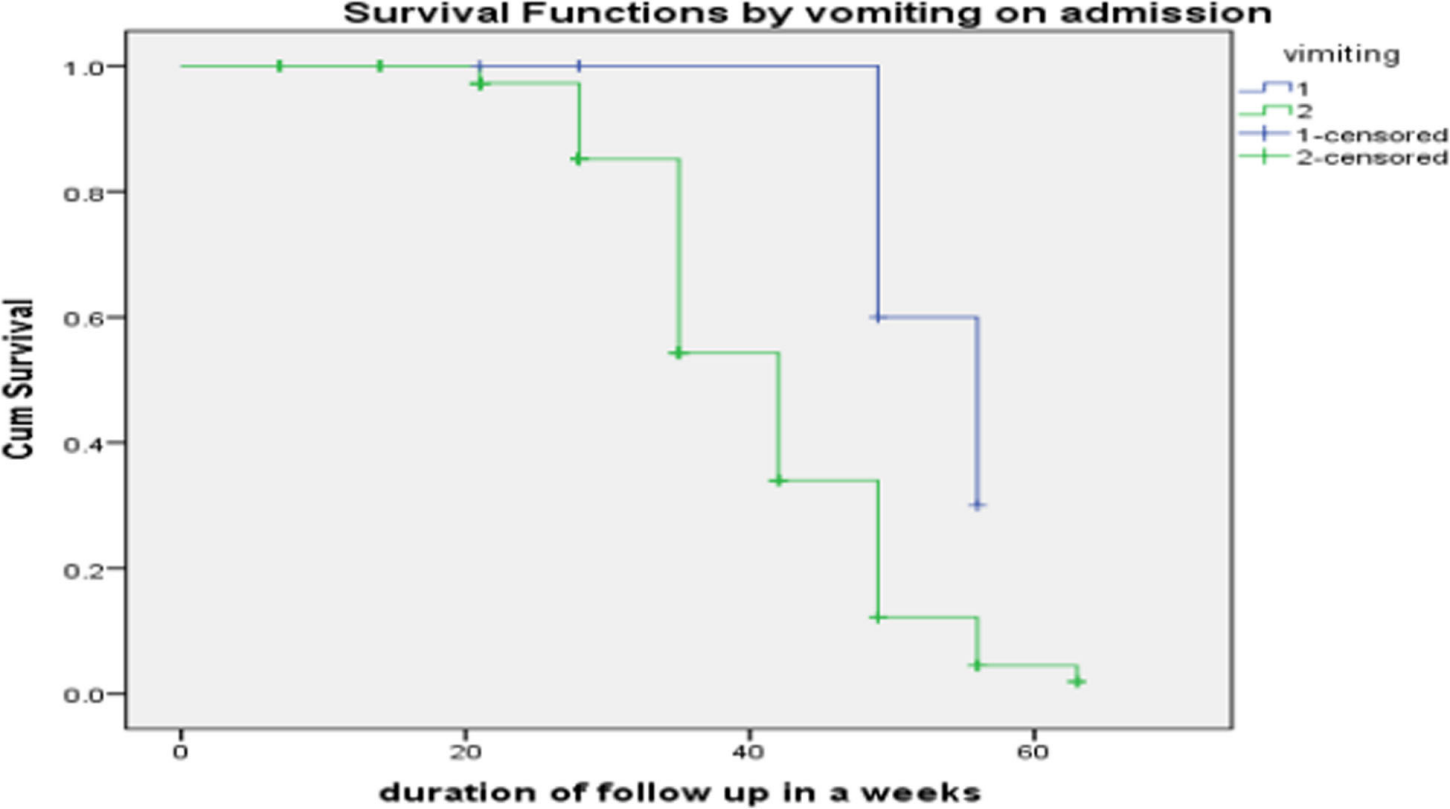
Kaplan-Meier curve of surviving SAM patients on time to recovery by vomiting on admission at OTP in north Gondar zone, northwest Ethiopia, 2017

Time to recovery for patients who had vomiting on admission a median recovery time was 56 days, whereas who did not have vomiting on admission had a median recovery times of 42 days and the difference was significant (*p*-value =0.038). Similarly, 60% of patients with vomiting still not recovered at 49 days as compared to 12.2% of patients without vomiting (Fig. 1.)

### Predictors of time to recovery among SAM patients

In the bi-variate analysis place of residence, sex, diarrhoea on admission, vitamin A, amoxicillin intake at admission, providing folic acid at admission, vomiting at admission and provision of deworming during follow-up were significantly associated with time to recovery. However, in the multi-variable Cox regression model diarrhea at admission, vomiting at admission, providing folic acid at admission, and provision of Amoxicillin at admission remained statistically significant predictors of time to recovery.

The hazard of patients with vomiting at admission prolonged time to recovery by 57% compared to those patients without vomiting AHR = 0.430 at CI 95%(0.205, 0.904). Similarly, the rate of time to recovery among who had diarrhea on admission delayed recovery time by 19% compared to patients who had no diarrhea (AHR = 0.811; 95%CI:0.735–0.993).

The rate of time to recovery among patients taken amoxicillin at admission was 2.304 times faster recover from SAM as compared to they did not took it (AHR = 2.304; 95%CI:1.680–3.161) (Table 6).

**Table 6:** Multi-variable Cox regression model among children with SAM at OTP in North Gondar zone, northwest Ethiopia, 2017(*n* = 389)

Variables	Outcome		CHR with 95%CI	AHR with 95% CI
	Event	Censored		
Residence				
Urban	175	78	1.247 (1.12,1.634)	0.837 (0.559, 1.25)
Rural	79	56	1	1
Sex				
Male	122	63	0.82(0.672,1.089)	0.845 (0.643,1.11)
Female	132	72	1	1
Diarrhea on admission				
Yes	51	17	0.80(0.682,0.997)	0.81(0.735, 0.99)*
No	203	118	1	1
Vomiting at admission				
Yes	10	2	0.473(0.25,0.894)	0.43 (0.205, 0.904)*
No	244	133	1	1
Amoxicillin intake at admission				
Yes	155	109	1.96 (1.504,2.544)	2.304 (1.680, 3.161)*
No	99	26	1	1
Folic acid at admission				
Yes	56	40	1	1
No	198	95	0.604(0.456,0.82)	0. 60 (0.399, 0.915)*
Vitamin A at admission				
Yes	35	19	1.348(1.114,1.54)	1.33 (0.841, 2.107)
No	219	117	1	1
Deworming during follow up				
Yes	7	1	1.426(1.237,1.73)	1.09 (0.386, 3.08)
No	247	134	1	1

* show statisticall significance association

## Discussion

This study assessed time to recovery of severely malnourished children aged 6–59 months managed on outpatient basis for a maximum duration of 8 weeks. The recovery rate was about 65.3%. This finding was lower than the sphere standard which states recovery rate should be greater than 75%.

The median length of stay was (38.5 ± IQR 14 days) for recovered/cured children and this study was found to be in line with a multicentre, randomized intervention study done in Mali which showed that median recovery time of the entire cohort was 42 days. Another prospective cohort study conducted in southern people, nation and nationalities region of Ethiopia showed that the median recovery time was 49 days [18, 19]. However, the finding of this study found to be lower duration of stay in the outpatient therapeutic program than 92 days length of time to recovery a study done in Afar, Ethiopia [20]. The difference showed because of giving a monthly supply of therapeutic food in an area when children enroll into OTP on admission and appetite test was not checked and inappropriate quantity or inadequate to their weight of RUTF provided to them. This leads to prolonged duration of time to recover from SAM.

This finding is supported by another study done from India with median time of 51 days with IQR of 5.6 days [21]. However, the finding of this study was found to be lower than a retrospective study conducted from Zambia with 24 weeks [22] and a prospective cohort study in rural Ethiopia with median time of 9 weeks and IQR of 4–15 weeks [23]. The difference could be due to no access to supplementary feeding, intervention protocol difference in a setting, differences discharge criteria.

Children who were taken amoxicillin as routine medicines on admission had better time to recovery as compared to those who were not taken the medication at admission. This is supported by study from Wolayita zone, Ethiopia [24]. However, findings from Kambata, south Ethiopia [25] showed that taking Amoxicillin had no difference in time to recovery among SAM patients.

Children with vomiting at admission complicate SAM and prolonged time to recovery. This finding is supported by the study conducted in Tigray, Ethiopia [8, 26].

Diarrhea at admission is a negative predictor of time-to-recovery from SAM. The time to recovery from OTP among children who had diarrhea during admission was delayed by 19%. This difference could be that diarrhea is linked to delay time to recovery and poor health outcomes of children with SAM. Similarly, the findings from rural Bangladesh suggest that diarrhea complicate and result in unfavorable nutritional consequences [27].

This study has its own strength and limitations. Since the study design was prospective follow up study we used a primary data and temporality issue was clearly ascertained. Therefore, cause and effect relationship is possible to establish. The response related to RUTF shared by other siblings or not might be affected by social desirability bias. Additionally, household and environmental factors of the care taker were not addressed. Other co-morbid conditions like Tuberculosis (TB), HIV, chronic conditions like cardiac and renal abnormality of the children were not assessed.

## Conclusions

The time to recovery has not met the minimum sphere international standard which was > 75% and a maximum of 8 weeks. Factors that prolong time-to-recovery include diarrhea and vomiting on admission. On the hand, routine medicines provision like amoxicillin on admission result in faster time to recovery from SAM.

## Data Availability

Data will be available upon request from the corresponding author.
